# Supplementary material for: Identification of the Role of TGR5 in the Regulation of Leydig Cell Homeostasis
Source: Int J Mol Sci. 2022 Dec 6;23(23):15398. doi: 10.3390/ijms232315398 (PMC9738292; doi:10.3390/ijms232315398)
Supplement: Supplementary file 1 [file ijms-23-15398-s001.zip › ijms-1974514-supplementary.pdf]

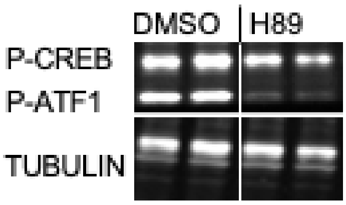

Figure S1. Representative western blots of P-CREB and TUBULIN in mLTC1 cells treated with vehicle (DMSO) or H89 for 2 hours.
